# Perturbation of the Human Microbiome as a Contributor to Inflammatory Bowel Disease

**DOI:** 10.3390/pathogens3030510

**Published:** 2014-06-30

**Authors:** Bayan Missaghi, Herman W. Barkema, Karen L. Madsen, Subrata Ghosh

**Affiliations:** 1 Department of Medicine, University of Calgary, Infection Prevention & Control, Alberta Health Services, Office 3685, 3500–26th Avenue Northeast, Calgary AB T1Y 6J4, Canada; 2 Department of Production of Animal Health, University of Calgary, HSC 2521, Health Sciences Centre, 2500 University Drive Northwest, Calgary AB T2N 1N4, Canada; E-Mail: barkema@ucalgary.ca; 3 Department of Medicine, University of Alberta, 7-142K Katz Building, Edmonton AB T6G 2E1, Canada; E-Mail: karen.madsen@ualberta.ca; 4 Department of Medicine, University of Calgary, Alberta Health Services, Room 930, North Tower, Foothills Medical Centre, 1403–29th St NW, Calgary T2N 4J8, Canada; E-Mail: subrata.ghosh@albertahealthservices.ca

**Keywords:** microbiota, metagenome, microbiome, inflammatory bowel disease, ulcerative colitis, Crohn’s Disease

## Abstract

The human microbiome consist of the composite genome of native flora that have evolved with humanity over millennia and which contains 150-fold more genes than the human genome. A “healthy” microbiome plays an important role in the maintenance of health and prevention of illness, inclusive of autoimmune disease such as inflammatory bowel disease (IBD). IBD is a prevalent spectrum of disorders, most notably defined by Crohn’s disease (CD) and ulcerative colitis (UC), which are associated with considerable suffering, morbidity, and cost. This review presents an outline of the loss of a normal microbiome as an etiology of immune dysregulation and IBD pathogenesis initiation. We, furthermore, summarize the knowledge on the role of a healthy microbiome in terms of its diversity and important functional elements and, lastly, conclude with some of the therapeutic interventions and modalities that are now being explored as potential applications of microbiome-host interactions.

## 1. Introduction

The human microbiome is the collective genome of indigenous microbiologic flora that has evolved with mankind over millennia. From the moment of birth, each part of the body becomes colonized by characteristic populations of organisms belonging to each of the three domains of life, Archaea, Bacteria and Eukarya [1,2]. However, the human gut microbiome is particularly distinct in that it is home to a plethora of bacteria, an estimated 100 trillion cells, which is approximately 10-fold more than the total number of human cells [3] and which encodes for 150-fold more unique genes than our own genome [4]. The majority of these bacteria cannot easily be cultivated using traditional culture techniques, and it has only been since the application of sequencing technology that the complement of human flora has been described in greater complexity [5]. Our species has co-evolved with these micro-organisms in a mutualistic fashion, and we have come to rely on them for many processes including immune system maturation, nutrition, maintenance of intestinal barrier permeability and function, and prevention of infection with pathologic species [3]. Indeed, the gut microbiota reside in close proximity to the luminal surface of the large and small bowel, placing them in near proximity to the immune system, and endowing them with a key role in immune function and maturation [6]. It has become increasingly clear that this fragile balance of organisms has many important associations with regards to the maintenance of health and the prevention of illness. It was our aim to review some of these concepts with a special emphasis on the role of the intestinal microbiota in inflammatory bowel disease (IBD).

It has been suggested that individuals can be categorized into three distinct enterotypes that are separated by dominance in the proportions of one of three genera: *Bacteroides* (enterotype 1), *Prevotella* (enterotype 2), and *Ruminococcus* (enterotype 3) [7]. These clusterings appear to have developed in response to primarily dietary environmental factors [7]. In this original study, the authors concluded that the robustness of these enterotypes across populations, as well as across phylogenetic and functional levels, was indicative of the fact that they appear to be the result of a limited number of well-balanced discrete bacterial community compositions [7]. However, more recent studies have demonstrated the existence of abundance gradients rather than distinct clustering, and have shown that the type of methodology used to examine microbiome composition can have significant effects on conclusions drawn [8].

## 2. Microbiome Alterations and Inflammatory Bowel Disease Immune Dysregulation

Inflammatory bowel disease is a chronic inflammatory disorder of the intestinal tract that is associated with abdominal pain, intestinal bleeding, weight loss, and diarrhea. The two most common forms of the condition include Crohn’s disease (CD) and ulcerative colitis (UC) [9]. These diseases are associated with considerable morbidity, and place a significant burden on individuals, families, and society, accounting for an estimated $1.8 billion of expense per year in Canada alone [10,11]. The cause of IBD remains unknown; however, it is thought to occur in genetically-predisposed individuals who are exposed to microbial, dietary, and environmental triggers [12]. The incidence of IBD has increased dramatically in developed countries over the past 50 years and has been linked with modernization and Western lifestyles [11]. Environmental risk factors are an essential component in the pathogenesis of IBD and are believed to be largely responsible for the rapid increase in world-wide incidence [12]. Please refer to Box 1.

**Box 1. Factors affecting the microbiome.**

- Age
- Genetics
- Diet
- Medications (antibiotics, *etc.*)
- Smoking status
- Obesity
- Pregnancy-status

In infants, gut microbial communities undergo radical compositional and functional changes in response to environmental exposures until a relatively stable community becomes established by approximately 2–3 years of age [13]. Establishment of the gut microbiome is influenced by early diet, antibiotic exposure, and method of birth, breast milk composition, and genetic background [14,15,16,17]. Antibiotic use during this period can significantly alter the composition of the gut microbiota and result in long term decreases in diversity and altered trajectories of immune development, which can modulate susceptibility to disease in later life [13,18,19]. Indeed, antibiotic exposure and infections during the first year of life have been identified as independent risk factors for IBD [20,21,22]. This is due to the fact that gut microbiota play an important role in immune regulation and barrier function of the gut, and that consequent alteration thereof may result in the loss of tissue integrity [23]. Both the innate and adaptive arms of the immune system have been shown to play a role in IBD, with altered adaptive immune function being the primary contributor to disease pathogenesis [6]. In general, this occurs mainly through increased pro-inflammatory cytokines driven by the T-helper subsets or by lack of effective anti-inflammatory regulatory T-cells [6].

Changes in innate immunity are also important and evidence exists that alterations in innate immune function are the primary driver of dysfunctional adaptive immune responses. It has been demonstrated that intestinal epithelial cells may directly or indirectly recognize luminal micro-organisms, and that toll-like receptors (TLRs) may be essential in the maintenance of epithelial cell homeostasis [24]. TLRs are specialized trans-membrane receptors of the interleukin-1 superfamily, which recognize and respond to pathogen-associated molecular patterns (PAMPS). The interactions between such TLRs and their ligands are involved in the clearance of pathogenic bacteria, leukocyte recruitment, epithelial homeostasis, and maintenance of barrier immunity. Many TLRs are upregulated in IBD once inflammation is established due to the increased presence of TLR promoter inducers, including inflammatory cytokines and NF-КB [24]. A loss of TLR signaling homeostasis in either direction, either an increase or decrease in activity, is thought to play a potential role in IBD pathogenesis [25].

Nucleotide-binding oligomerization domain-containing protein 2 (NOD2), also known as caspase recruitment domain-containing protein 15 (CARD15), is a protein which plays an important role in immune system functioning via recognition of bacterial molecules (such as peptidoglycans) and stimulation of appropriate responses. NOD2 alleles are mutated in approximately 15% of CD patients, and this can result in defective neutrophil recruitment in these patients as a result of NOD2 and interleukin 8 (IL-8), and NOD2 and TLR receptor pathway interactions [26]. This supports the concept that the adaptive immune dysfunction in CD may occur due to a failure to receive an early priming signal to pathogenic micro-organisms, resulting in a failure to clear them and the development of abnormal subsequent immune responses to their antigens. Studies now suggest that CD patients with abnormal NOD2 have an increased susceptibility to the generation of strongly polarised Th1 responses, resulting in an overly aggressive adaptive immune response to common intestinal flora and thereby setting the stage for IBD [27].

The general concept of immune dysregulation has led credence to the “hygiene hypothesis”, a theory that has been advanced as a possible explanation for the increased incidence of IBD, which claims that improvements in hygiene—particularly in developed countries—are congruent with a rise in autoimmune illnesses [9,11]. Strachan (1989) was the first scientist to connect this hypothesis to an increase in allergic diseases and believed that a child could be overly protected from exposure to microbes with lack of exposure to these agents, in early childhood, resulting in more widespread clinical expression of atopy [28] due to immune aberrancy. It is for this reason that many scientists now advocate for a moderate approach towards cleanliness in young children [29].

## 3. Microbiome Bacterial Dysbiosis in IBD Pathogenesis

A growing body of literature implicates the abnormal overgrowth or dominance of particular bacterial species, at the expense of others, in the pathogenesis of IBD. Notably, mouse model studies of IBD have shown protection against the development of IBD in a germ-free environment, and lend credence to the role of gut flora in the pathogenesis of this spectrum of illnesses [30]. Furthermore, other studies have revealed that the intestinal flora of patients with IBD have less bacterial species diversity and less stability over time, suggestive of an association between alteration of the human microbiota and development of this type of disease [31]. In healthy hosts, *Firmicutes*, felt to contribute in a key fashion to micronutrient metabolism, are amongst the most dominant constituents of the human indigenous gut microflora, accounting for the largest proportion (75%) of bacteria [2]. Several studies to date [32,33,34] have demonstrated reduced *Firmicutes*, in particular *Faecalibacterium prausnitzii* and *Roseburia,* and increased *Ruminococcus* and *Enterobacteriaceae*, especially adherent invasive *Escherichia coli* (AIEC), in IBD [35,36,37,38,39,40,41,42,43,44,45].

Some research to date has demonstrated an increased prevalence of *Escherichia coli* in the ileum of CD patients [36], and a very recent study has shown the ability of *E. coli* to trigger and potentiate intestinal inflammation in mouse models [46]. Despite, the association of this particular organism with IBD and, in particular, CD, the precise role of adherent-invasive *E. coli* (AIEC) in the initiation or activation of IBD is not yet fully defined. However, there is evidence for upregulation of microRNAs, which reduce expression of proteins necessary for the autophagy response in intestinal cells [47]. This suggests that such infected epithelial cells are prevented from self-degradement and recycling of cell components. One theory is that AIEC strains, having the capability of adhering to and invading both intestinal epithelial cells and macrophages, can translocate across the bowel wall and continuously activate further macrophages, leading to the formulation of granulomas [36]. It is known that micro-organisms that are able to penetrate the bowel epithelial barrier and to bypass macrophage killing can trigger a powerful and long lasting inflammatory response [48,49].

Other research has reported an interesting increased prevalence of other organisms, including *Mycobacterium avium* subspecies *paratuberculosis* (MAP) in the intestinal tissue of CD patients [50,51]. MAP has been defined as the etiology of Johne’s disease in cattle, a chronic granulomatous illness that is clinically and pathologically similar to CD in humans [52]. Moreover, several studies have revealed that a high proportion of CD patients are infected with MAP compared to persons not having IBD [53]. In fact, a fairly recent imputation-based association analysis was quite conclusive in the overlap and correlation shown between susceptibility loci for CD and infection with MAP [54]. Unfortunately, however, no research to date has either confirmed or denied the precise role of any particular organisms in IBD. In fact, the source of MAP in humans is felt by some researchers to be either an environmental or zoonotic epiphenomenon. A two-year study by Selby *et al.* revealed that empiric therapy typically effective against MAP, with clarithromycin, rifabutin, and clofazimine for up to two years, did not provide sustained benefits [55]. On the other hand, using the data from this study, one can also refute the conclusions drawn, noting that the antibiotic arm did significantly better than the control arm for as long as antibiotics were administered [56]. This is suggestive that MAP is a significant player in at least a subset of IBD patients, and that this type of therapy may be effective for them. Further study will be necessary to sort out this controversy.

An emerging theory is that a dysbiosis or imbalance of intestinal flora may be a trigger for IBD in those who may be susceptible, a problem that is normally avoided through the maintenance of a healthy microbiome. There are many reasons why the presence of normal healthy commensal organisms prevents this process from occurring. One is that commensal microbes resist colonization by enteric the pathogens listed above, both directly and indirectly [57]. There are several ways in which this is achieved. Firstly, the most basic method in which normal microbiota favour resistance against opportunistic infection is via niche competition. This involves competition for sites of colonization and nutrient uptake at host epithelial surfaces [58], and modulation of protective immune responses [57]. Another modality by which commensal organisms help resist colonization by bacteria associated with IBD is by promotion of barrier immunity. An example of this would be induction of epithelial production of mucin, secretion of mucosal immunoglobulins and the expression of antimicrobial peptides via host TLR recognition of PAMPs [57] as alluded to previously. Yet a third modality of pathogen resistance relates to the anaerobic environment of the bowels, a factor which allows particular gut commensals to harness energy via fermentation of nutrients passing through the lumen, resulting in the production of short chain fatty acids (SCFAs). SCFAs include acetate, butyrate and propionate, and are resistant starches that are generally not well digested in the upper GI tract [59]. SCFAs strengthen the intestinal epithelium by increasing tight junction protein (TJP) production and by increasing transepithelial electrical resistance (TEER) [2] as well as by affecting the immune system directly [60]. Thus, it is clear that diet, too, by selecting for particular micro-organisms and by resulting in specific metabolites, has a profound impact on the selection of existing normal intestinal flora.

## 4. The Microbiome, Obesity and CD

The worldwide obesity epidemic has more than doubled since 1980, and this condition is associated not solely with metabolic disorders, including type 2 diabetes and cardiovascular disease, but also with a wide variety of disorders such as cancer, sleep apnea, osteoarthritis and gallbladder disease [61]. Though the present view is that the major etiology of obesity is unbalanced energy intake and expenditure in association with genetic predisposition, environmental factors may also play a role. Amongst the important outside factors impacting the host’s response to diet is the gut microbiome [62]. The initial demonstration of particular alterations in composition between the intestinal flora of obese and lean phenotypes was made in leptin-deficient mice, in which the guts of obese mice contained fewer *Bacterioidetes* and increased *Firmicutes* than other mice [63]. Subsequent studies revealed that the transfer of obese mouse gut flora to germ-free mice could reproduce the donor obese phenotype in the recipient mice [64], and several recent studies, using pyrosequencing and advanced PCR techniques, have demonstrated that high-fat diets increase the *Firmicutes/Bacterioidetes* ratio and decrease *Bifidobacterium* spp. [65]. Moreover, a higher abundance of *Ruminococcaceae* and *Rikenellaceae* was seen in leptin-resistant obese and diabetic mice than their slim counterparts [66]. Of some interest, it has also been shown that bacterial ATPase complexes correlate strongly with human BMI, further supporting the association between the intestinal flora’s capacity for energy harvest and obesity in the host [67]. Moreover, adipocytes have been demonstrated to release a variety of proinflammatory cytokines and peptides, and it is believed that visceral adiposity may play an important role in the commencement and maintenance of inflammation in CD [68]. Based on a time-trend analysis from data collected between 1991 and 2008, a clear correlation has been shown between increasing weight over time and CD [68].

## 5. Human Gut Microbiome Diversity and Functional Changes

It has recently been discovered that the evolutionary composition of gut bacterial flora, regardless of geography, evolves towards an “adult” phenotype by the third year of life [69]. Furthermore, regardless of geography, interpersonal variation in gut flora is greater between children than between adults [69]. Moreover, microorganism diversity increases with age, and there are significant phylogenetic differences amongst populations living in different countries and regions [69]. Many of these differences are due to diet and the availability of particular nutrients in the environment with increases in proportions of various bacterial species found to be primarily related to functional requirements. For example, individuals living in rural Africa and South America have gut microbiomes that are more similar to those of herbivorous animals, whereas Western microbiomes are more reflective of those of carnivores [70]. Thus, these are clear adaptations determined in response to varying diets in these regions.

Over the past several years some interesting trends were discovered in regards to functions of gut micobiota. Though some studies have shown clear variability in the composition of human microbiota over time, the functional potential of the microbiome remains strikingly stable [71]. Using metagenomic approaches, it has been shown that 12% of gut-related metabolic pathways are different between IBD patients and healthy controls versus only 2% of genus-level clades in this context [72]. In keeping with taxonomic profiling studies revealing lessening numbers of Firmicutes, one metagenomic trend that has been shown is that there is a reduction in butyrate and propionate metabolism genes in CD [72], as well as lower levels of other SCFAs [43]. There is also an increase in functions associated with auxotrophic bacteria, including a decrease in amino acid biosynthesis, and an increase in amino acid transfer [72]. Given the presence of tissue destruction in inflammatory conditions, these bacteria tend to thrive in such conditions where nutrients are easily accessible in the environment [72]. Furthermore, studies have shown an increase in sulphate-reducing bacteria in IBD, an interesting fact when one considers that a mechanism of a common medical treatment for IBD, mesalamine, is the inhibition of fecal sulphide production [73]. Genes that take part in the metabolism of the sulfa-containing amino acid cysteine are increased in IBD, as is increased sulphate transport [72].

## 6. Impact of Cigarette Smoking on the Microbiome

Other environment factors also impact the gut microbiota. Smoking is an important risk factor in IBD pathogenesis with most studies showing a protective effect of cigarette smoking in UC and a deleterious effect in CD [74,75]. Smoking cessation induces prominent alterations in the composition of normal intestinal flora [76]. After cigarette cessation, comparable shifts have been seen in flora composition as have been described in obese *versus* lean humans and mice [63,77]; specifically, an increase in the proportion of *Firmicutes* relative to *Bacterioidetes*. Moreover, a concurrent mean weight gain of 2.2 kg occurred during the observation period of these mice, in the absence of alterations in total calorie intake or other dietary changes [76], suggesting a role of commensal organism changes and consequent metabolic alterations in pathogenic weight gain post smoking cessation. However, the precise molecular and cell effects of smoking and smoking cessation on gut microbiota continue to be relatively poorly understood and are an area for further detailed study.

## 7. Impact of Antimicrobial Therapy on the Microbiome

It is well known that, since the 1950s, antibiotics have been liberally used in the agricultural industry as growth promoters, though the mechanism for this effect has been poorly understood. Notably, in Canada and the U.S., the heaviest use of such antimicrobials is within farms, with restricted doses fed to livestock in order to increase their growth by up to 15% [78,79]. Interestingly, these effects have shown to be relatively consistent across a broad variety of vertebrate species, including mammals (cattle, swine, sheep) and birds (chickens, turkeys), and follow oral administration of these antibiotics, suggesting that the gastrointestinal microbiota may be a notable target [80]. Moreover, the fact that these effects are not agent specific and have been shown true for macrolides, tetracyclines, pencillins, and other agents, indicates that these growth effects are not side effects associated with a particular class of antimicrobials. Within this context, it has been postulated that sub-therapeutic administration (STA) of such molecules may modify the structure of the gut microbiome and its metabolic function to a significant extent [80]. In fact, we know well that antibiotics can have a great effect on the composition of the microbiome in comparison to other environmental factors. As alluded to earlier, antibiotics administered within the first year of life are believed to cause atrophy of the microbiome and to increase the risk of future autoimmune disease as a consequence [20].

Interestingly, murine model studies have confirmed that STA of antimicrobials can both increase adiposity in mice, as well as increase hormone levels that play a role in metabolism by altering the proportions of various microbiota. Specifically, one recent novel study showed that an increase in relative concentrations of Firmicutes compared to Bacterioidetes in mice treated with STA of antibiotics [80]. Perhaps even more interestingly, STA of these molecules were shown in this study to stimulate adipogenesis by increasing SCFA levels and, thereby, by providing direct energy to colonocytes [80], confirming some of the functional pathway alterations that occur as a result of antimicrobial administration. This loss of equilibrium and homeostasis may become substantial when prolonged, and may result in changes in host health and subsequent disease [81]. Indeed, murine studies are currently being explored to define models for a variety of diseases related to microbiome shifts and alterations as a result of antimicrobial use.

## 8. Therapeutic Approaches and Applications of the Microbiome

Though antimicrobials have a well-established role in the treatment of septic complications of IBD, their benefit in treating the underlying disease process is more controversial. A number of controlled trials and observational studies have been published over the last 30 years regarding the use of antibiotics in treating CD [82,83,84]. Most of these are small studies of short duration and with significant methodological disadvantages, including the use of different endpoints and outcomes. However, a 2011 meta-analysis of randomized controlled trials revealed superiority of antibiotics to placebo in the induction of remission of active CD as well as in the maintenance of remission [85]. Another meta-analysis of 10 randomized trials in 2012 demonstrated an improvement in symptoms in IBD patients treated with antibiotics as opposed to placebo [86]. Regarding UC, the 2011 meta-analysis showed a modest benefit in induction of remission of active disease via antibiotics relative to placebo [85], though less promising than in CD. In reference to the study by Selby *et al.*, though anti-tuberculosis treatment has shown some benefit in CD, it is unclear whether this is as a result of the treatment of MAP, the treatment of other commensal organisms or via other nonspecific anti-inflammatory effects [55]. In terms of the choice of antimicrobial regimen, though there is significant variation and little standardization in the literature, most would advocate for the use of a combination of ciprofloxacin and metronidazole based primarily on clinical experience.

Probiotics are “live micro-organisms which, when administered in adequate amounts, confer a health benefit on the host” [87]. Indeed, several studies in mouse models have demonstrated prevention of the onset of colitis and reduction of inflammation in established disease using *Lactobacillus* spp. and mixed culture supplementation [88,89,90]. However, clinical data on the use of probiotics in humans is limited and most studies have not been placebo controlled [91]. Moreover, more study is needed on the properties of different bacterial strains for various indications, as well as information on combinations, dosage and duration of therapy. A related intervention involves the use of prebiotics, food ingredients that are not digested or absorbed in the upper GI tract and which are fermented in the lower GI tract, promoting the growth of micro-organisms with beneficial effects. For instance, inulin and oligofructose are fermented in the large intestine and encourage the growth of native lactobacilli and bifidobacteria among other organisms, effects which are associated with reduced mucosal inflammation in animal models of IBD [92].

Fecal microbiota transplantation (FMT) is a procedure, which has rapidly grown in acceptability and popularity over the past decade. In the animal kingdom, coprophagy (consumption of feces) is quite common and observed in a variety of species [93,94]. The practice of transfaunation, the transfer of gastrointestinal contents, between animals has been practiced for centuries by veterinarians and, whether conducted naturally or artificially, contributes to accelerated maturation of the gut, enhanced digestion of nutrients and increased resistance to colonization of the gut by pathogenic organisms [95]. Historically, despite an innate repugnance for stool, human civilization has used variations of FMT for thousands of years. The earliest known documentation of this comes from Chinese medicine, referring to the use of various fecal preparations for the treatment of human gastrointestinal illnesses [96]. However, there are also examples of such practice in other parts of the world, including Europe in the late 1600s, where Dr. Franz Paullini, a German physician, published a book on the uses of human and animal feces entitled *Hailsame Dreck-Apotheke* (Salutary Filth-Pharmacy) [95].

In modern times, FMT has been hailed as an extremely effective therapeutic procedure for treatment refractory *Clostridium difficile* infection (CDI) [97]. At this point in time, the FDA is allowing the use of FMT for CDI that remains refractory to other therapeutic modalities. Given the success of FMT for this indication, it would be sensible to believe that this procedure or derivatives thereof may eventually be applied to several other medical conditions on a more regular basis, including IBD. Some researchers now advocate for the streamlining of FMT by such interventions as “auto-banking,” in which a sample of patients’ stool is collected and frozen on admission to an inpatient facility for later implantation after completion of antimicrobial therapy [98]. Though theoretically reasonable, such methods will certainly require further study and approval by regulatory bodies.

In the context of increasing evidence of the importance of our normal microbiota for the maintenance of health and prevention of disease, there has been a recent explosion of interest in modulating the microbiome to restore balance and promote health. One such application, a derivative of FMT, has been labelled as “Microbial Ecosystem Therapeutics” (MET) and involves the replacement of a damaged and/or dysfunctional microbiota with a fully developed and “healthy” constellation of intestinal bacteria in a synthetic fashion [99]. As opposed to traditional probiotic therapies, which involve the use of a single strain or a few strains of bacteria, MET involves the application of entire intestinal bacterial populations, which more closely resemble our own normal flora. The potential implications for such therapy are vast and include the treatment of CDI in addition to IBD, irritable bowel syndrome (IBS), obesity and metabolic syndrome, and even certain types of autism [99].

However, it should be noted that at present the use of FMT or MET for non-CDI indications is an area of intense study and that the optimal approaches appear unclear. Despite anecdotal reports of successful treatment of UC and CD [100,101], others, including Kump *et al*., have reported no clinical improvement in small series of patients with UC [102]. It is clear that this is a ripe area for further research.

Moreover, SCFAs, the main metabolic products of anaerobic fermentation in the bowel, have been recognized as important mediators in intestinal epithelial barrier permeability and immune function [60]. Indeed, higher concentrations of such fatty acids in the intestines and in the blood have a direct impact on reduction of predisposition to or intensity of inflammatory conditions, including IBD and cancer [60]. In this context, many researchers are now considering the potential therapeutic applications of SCFAs and their derivatives in the treatment of IBD [103,104,105]. Though results have been quite positive overall, further study is necessary to establish the dose, frequency and form of SCFA products to receive ideal results. Yet another implication of this research is the use of potential SCFA receptor agonists to achieve desired effects in terms of strengthening intestinal barrier permeability and priming the immune system.

Though the use of antimicrobials leading to selection of multi-drug resistant organisms (MDROs) is a growing cause for concern, many researchers would now argue that the likelihood of such medications to disturb indigenous flora, facilitating the spread of prior selected resistant strains or their resistant traits, is even more important [98]. This pressure on the microbiome will, of course, not only impact individual patients, but the whole of society as MDROs continue to spread as a result. Several important studies have now demonstrated that our current antibiotics, though effective as anti-infective agents, can cause significant collateral harm to our microbiome [106,107]. In this context, another important application of the microbiome in therapeutics involves studying the impact that each of our current antimicrobial classes makes in this realm, and to put stewardship measures in place to favour the use of drugs that have a low microbiome damage profile. This approach could be further expanded to the preclinical drug approval process to ensure that our drugs in development have the lowest possible impact on the status of our normal commensals [107]. Not only will this aid in improving the outcomes of individual patients, but will also help with infection control efforts to prevent and/or shorten the duration of MDRO colonization.

Furthermore, other researchers have advocated for the use of specific medications to be given after an antimicrobial course is complete in order to break down or bind any residual drug that will only serve to further damage the microbiome [108,109]. Indeed, there is a time lag of several weeks to months (and sometimes longer) between the final administration of antimicrobials and reconstitution of the microbiome to pre-treatment levels. This delay, in animal models, is linked with an increased propensity to secondary infections [110].

As we look to the future and as more knowledge is gained regarding host-microbiome interactions and the biochemical and immunologic interactions that lead to the release of antimicrobial peptides and maintenance of barrier immunity, desired effects may be achieved through novel modalities and approaches. Perhaps, we may one day manipulate the host into producing the peptides produced by an intact and healthy microbiome through the application of bacterial antigens or, better yet, the administration of purified peptides and other protective molecules without the need for application of live micro-organisms [98]. This will allow healthcare providers to artificially assist with healing the host intestinal barrier without subjecting the patient to the fecal transplant-associated risk of pathogen transmission and will avoid the inherent aversion to receiving a FMT.

## 9. Conclusions

Environmental factors, including the microbial composition of the gut, may be as important as genetic determinants in the pathogenesis of IBD, such as CD and UC. However, many details are yet to be determined. Despite this, it is clear that IBD disease pathogenesis involves a combination of an aberrant immune system along with dysbiosis in the gut microbiome. Further study and a better understanding of important host-microbial interactions will allow us to accelerate our development of novel therapeutic applications, such as FMT and MET, the use of innovative scoring systems for the impact of antimicrobials on the microbiome, and the hopeful eventual use of purified peptides and other molecules. The ultimate goal will be to optimally restore the normal and healthy mutualistic balance between the host and microbiota, which has existed for millennia.
